# Supplementary material for: Composition-, Size-, and Surface Functionalization-Dependent Optical Properties of Lead Bromide Perovskite Nanocrystals
Source: J Phys Chem Lett. 2020 Feb 23;11(6):2079–85. doi: 10.1021/acs.jpclett.0c00266 (PMC7997568; doi:10.1021/acs.jpclett.0c00266)
Supplement: Supplementary file 1 — jz0c00266_si_001.pdf [file jz0c00266_si_001.pdf]

Supporting Information for

Composition, Size and Surface Functionalization dependent Optical Properties of Lead Bromide Perovskite Nanocrystals

Palvasha Ijaz,[1,2] Muhammad Imran,[1] Márcio M. Soares,[3] Hélio C. N. Tolentino,[3] Beatriz Martín-García,[1,4] Cinzia Giannini,[5] Iwan Moreels,[1,6] Liberato Manna*,[1] Roman Krahne*[1]

[1]*Department of Nanochemistry,* [4]*Graphene Labs, Istituto Italiano di Tecnologia, Via Morego 30, 16163 Genova, Italy*

[2]*Dipartimento di Chimica e Chimica Industriale, Università degli Studi di Genova, Via Dodecaneso 31, 16146 Genova, Italy*

[3]*Brazilian Synchrotron Light Laboratory (LNLS), Brazilian Center for Research in Energy and Materials (CNPEM), Campinas, SP, 13083-970, Brazil*

[5]*Istituto di Cristallografia - Consiglio Nazionale delle Ricerche (IC-CNR), via Amendola 122/O, I-70126 Bari, Italy*

[6]*Department of Chemistry, Ghent University, Krijgslaan 281-S3, 9000 Gent, Belgium*

Materials

Lead acetate trihydrate (($PbAc_2{\cdot}3H_2O$), 99.99%), cesium carbonate (Cs_2CO_3, reagent Plus, 99%), formamidinium (FA) acetate, methylamine (2 M solution in tetrahydrofuran, THF), benzoyl bromide (C_6H_5COBr, 97%), ethyl acetate (98.8%), toluene (anhydrous, 99.5%), didodecyldimethylammonium bromide, oleylamine (70%), octadecene (ODE, technical grade, 90%), and oleic acid (OA, 90%), were purchased from Sigma-Aldrich. Didodecylamine (DDDAm, 97%), was purchased from TCI. All chemicals were used without any further purification unless stated otherwise.

Synthesis of $APbBr_3$ (A=Cs, MA, FA) NCs: First generation of $APbBr_3$ NCs were prepared using typical ligands such as oleic acid and oleylamine following a previously reported procedure.[1]

i) Synthesis of $CsPbBr_3$ NCs, 76 mg of lead (II) acetate trihydrate, 16 mg of cesium carbonate, 0.3 mL of oleic acid, 1.0 mL of oleylamine and 10 mL of ODE were combined in a 25 mL 3-neck flask equipped with a thermocouple and a magnetic stirrer. The reaction mixture was degassed for 5 min at room temperature and then for one hour at 120 °C. Next, the temperature was increased to 175 °C and a solution of benzoyl bromide (70 µL) in ODE (500 µL, which had previously been degassed for an hour at 120 °C and stored in glove box) was swiftly injected. The reaction mixture was immediately cooled down using an ice-water bath. Thereafter, 5 mL of toluene was added to the crude solution, then it was centrifuged at 5000 rpm for 10 min. The supernatant was discarded, and the precipitate was redispersed in 5 mL of toluene.

ii). Synthesis of $MAPbBr_3$ NCs: 76 mg of lead (II) acetate trihydrate, 2.5 mL of OA, 0.025 mL of OLAM, and 5 mL of ODE were mixed in a 25 mL 3-neck round-bottom flask and dried under vacuum for 1 h at

120 °C. Next, the temperature was lowered to 65 °C and a methylamine solution (0.170 mL) was injected, followed by the swift injection of benzoyl bromide solution (70 µL) in ODE (500 µL, which had previously been degassed for an hour at 120 °C and stored in glove box). The reaction mixture was quenched by the addition of 5 mL of toluene after 30 s and NCs were collected by centrifuging the crude solution at 4000 rpm for 10 min. The supernatant was discarded, and the precipitate was redispersed in 5 mL of toluene.

iii) Synthesis of $FAPbBr_3$ NCs: 76 mg of lead (II) acetate trihydrate, formamidinium acetate (40 mg), 2.5 mL of OA, 0.025 mL of OLAM, and 5 mL of ODE were mixed in a 25 mL 3-neck round-bottom flask and dried under vacuum for 1 h at 120 °C. Then, the temperature was lowered to 75°C under N_2, and a solution of benzoyl bromide (70 µL) in ODE (500 µL, which had previously been degassed for an hour at 120 °C and stored in glove box) was swiftly injected. After 30 s, the reaction mixture was cooled down in an ice−water bath. Thereafter, 5 mL of toluene was added to the crude solution, then it was centrifuged at 5000 rpm for 10 min. The supernatant was discarded, and the precipitate was redispersed in 5 mL of toluene for further use.

Synthesis of Cs-Oleate capped $CsPbBr_3$ NCs:

Cs-oleate capped $CsPbBr_3$ NCs were synthesized following our previously reported procedure using standard Schlenk line techniques.[2] Briefly, lead (II) acetate trihydrate (76 mg) cesium carbonate (16 mg) and ODE (10 mL) were combined in a 25 mL 3-neck flask equipped with a thermocouple and a magnetic stirrer. The reaction mixture was degassed for 5 min at room temperature and then for one hour at 115 °C. Then, a ligand mixture containing oleic acid (1.5 mL, previously degassed for an hour at 120 °C and stored in glove box), didodecylamine (1.25 mmol, 443 mg) dissolved in 1 mL of anhydrous toluene was rapidly injected under nitrogen. After the complete dissolution of the metal precursors, the temperature was decreased to 70 °C and a solution of benzoyl bromide (50 µL) in anhydrous toluene (500 µL) was swiftly injected. After 60 s, the reaction mixture was cooled down by using a water bath. Then 20 mL of an ethyl acetate and toluene mixture (with a ratio of 6:1) was added into the crude solution to destabilize the colloids followed by centrifugation at 6000 rpm for 10 min. Finally, the supernatant was discarded, and the precipitate was redispersed in toluene.

Synthesis of QAB capped $CsPbBr_3$ NCs: QAB capped $CsPbBr_3$ NCs were prepared following a previously reported ligand exchange strategy.[3] Briefly, Cs-oleate capped NCs were prepared following above mentioned procedure[2] and the crude reaction mixture containing the $CsPbBr_3$ NCs (3 mL) was treated with an anhydrous toluene solution containing the didodecyldimethylammonium bromide salt (2 mL, 0.025M) and the mixture was vigorously stirred for 1 min. Thereafter, the NCs were washed by addition of 15 mL of ethyl acetate followed by centrifugation at 6000 rpm for 10 min and re-dispersion in toluene.

Transmission Electron Microscopy (TEM): Bright field TEM images were acquired on samples prepared by drop-casting diluted colloidal solutions on carbon film-coated 200 mesh copper grids, using a JEOL-1100 microscope operating at an acceleration voltage of 100 kV.

Steady-state UV-VIS Absorbance and Photoluminescence: Absorbance and photoluminescence spectra of dilute toluene dispersions in quartz cuvettes (1-cm path length) were recorded using a Varian Cary 300 UV-VIS spectrophotometer. The photoluminescence spectra were acquired using an excitation wavelength of 350 nm.

NC film preparation: Colloidal dispersions with a concentration of NCs of 18±2 mg/mL were used to prepare NCs films. Briefly, each sapphire substrate was washed with acetone and dried with compressed air. Then, 30 uL of NCs dispersion was drop-cast on the sapphire substrate and the solvent was allowed to evaporate.

Temperature dependent Photoluminescence Measurements: Steady-state PL spectra and time resolved PL (TRPL) lifetime were recorded with an Edinburgh Instruments fluorescence spectrometer (FLS920) coupled to an optical fiber. The nanocrystal films deposited on a sapphire substrate (TedPella®) were placed inside a closed-cycle helium cryostat (Advanced Research Systems, Inc.). The steady-state PL was collected by exciting the sample with a 405 nm laser diode at 50 ns repetition period. The PL decay traces were recorded at the PL peak position by exciting the sample at 405 nm using a 50 ps laser diode at a repetition rate of 0.5-200 MHz and an emission bandwidth of 1-5 nm.

Temperature dependent X-ray Diffraction (XRD): The XRD data was collected at the XPD beamline of the Brazilian Synchrotron Light Laboratory (LNLS), in the temperature range from 300 K down to 20 K using a He closed cycle cryostat. Measurements were performed at 10 keV energy (λ=1.23984 Å) in

the Bragg-Brentano geometry using a 1D Mythen detector. The instrumental function has been determined from a NIST Al_2O_3 standard.

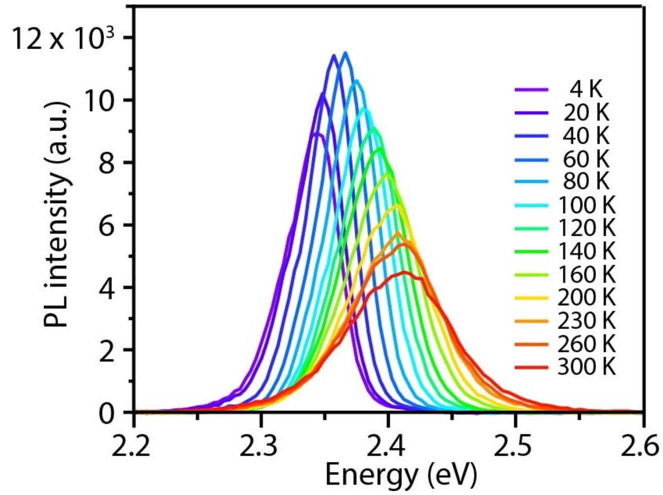

Figure S1. Photoluminescence (PL) spectra measured from 4 K to 300 K for mixed ligands capped $CsPbBr_3$ NCs.

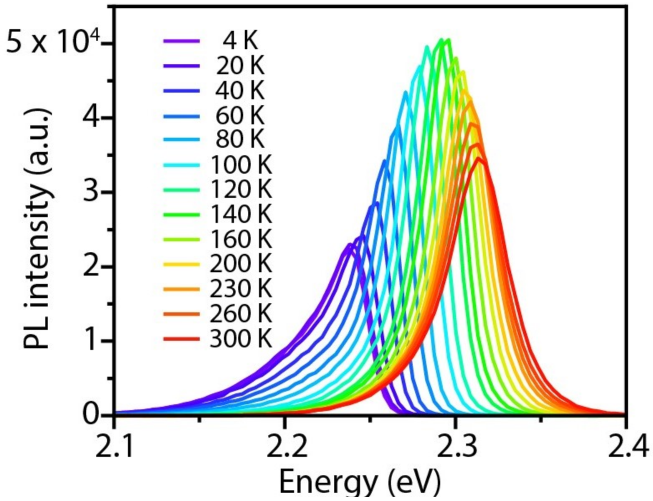

Figure S2. PL spectra measured from 4 K to 300 K for mixed ligands capped $MAPbBr_3$ NCs.

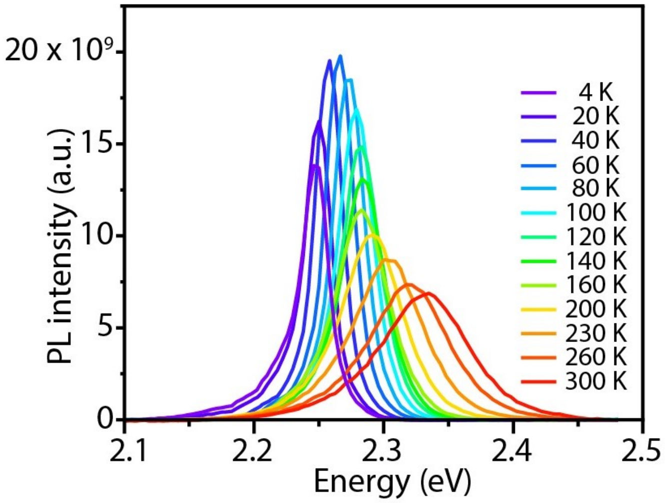

Figure S3. PL spectra measured from 4 K to 300 K for mixed ligands capped $FAPbBr_3$ NCs.

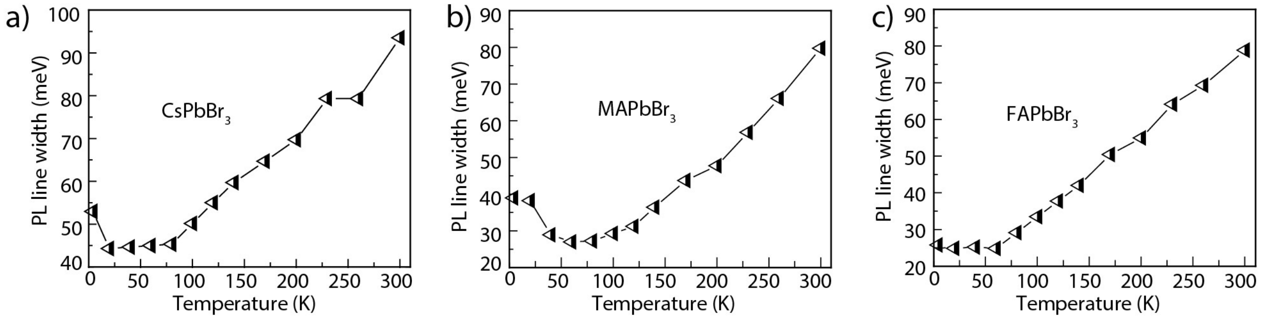

Figure S4. Evolution of PL line width as a function of temperature for mixed ligand capped (a) $CsPbBr_3$, (b) $MAPbBr_3$ and (c) $FAPbBr_3$ NC films.

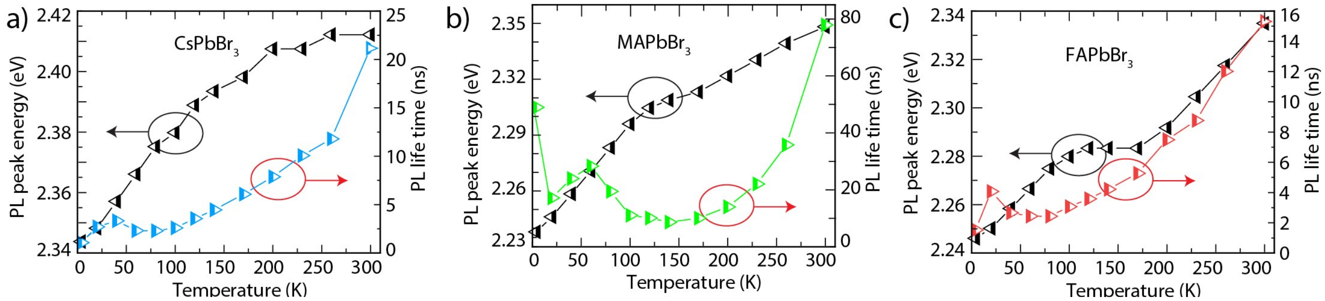

Figure S5. PL peak energy and PL lifetimes at elevated temperatures for mixed ligand capped (a) $CsPbBr_3$, (b) $MAPbBr_3$ and (c) $FAPbBr_3$ NC films.

Table S1. Parameters of the exponential fitting of the PL decay traces, and the average PL lifetimes of $CsPbBr_3$ NCs obtained from the traces in Figure 2c:

Temperature (K)	A_1	τ_1 (ns)	A_2	τ_2 (ns)	A_3	τ_3 (ns)	τ_{AVG} (ns)
4	13689	0.56	1366	2.32			1.08
20	12185	0.75	548	7.23			2.71
40	9474	0.92	3744	2.56	456.54	9.74	3.33
60	10691	1.20	4217	2.27	320.89	8.01	2.31
80	10385	1.64	3425	1.60	829.84	5.56	2.30
100	9666	1.94	2713	1.47	1998.24	4.48	2.60
120	9597	2.45	1289	4.81	234.50	10.83	3.56
140	9773	3.30	171	7.38	612.63	9.98	4.46
170	9561	4.41	159	10.32	954.33	11.73	6.08
200	7983	5.08	1333	10.91	2096.93	10.89	7.87
230	5950	5.17	3107	10.39	2868.11	13.65	10.07
260	6335	6.61	2634	14.66	2752.49	14.47	11.80
300	4735	6.43	3485	27.09	4053.17	19.75	21.15

Table S2. Parameters of the exponential fitting of the PL decay traces, and the average PL lifetimes of $MAPbBr_3$ NCs obtained from the traces in Figure 2c:

Temperature (K)	A_1	τ_1 (ns)	A_2	τ_2 (ns)	A_3	τ_3 (ns)	A_4	τ_4 (ns)	τ_{AVG} (ns)
4	10532	0.68	2635	3.22	528.63	21.55	97	131.00	48.97
20	9835	1.04	2376	4.11	224.6	22.67	14	144.48	17.16
40	10128	1.17	1554	5.66	204.98	28.10	18	159.30	23.96
60	9799	1.39	1769	6.28	278.97	29.14	21	182.59	28.33
80	9608	1.82	1754	8.03	197.52	32.75	14	173.26	19.47
100	7468	1.35	5266	4.26	1007.19	13.95	55	67.24	11.04
120	8518	3.94	2082	11.87	20.52	94.11			10.09
140	1669	2.85	5147	6.94	351.81	20.78			8.65
170	1096	2.74	6341	8.21	676.40	19.13			10.05
200	1689	4.13	6252	12.67	480.86	27.39			14.02
230	1527	4.86	6464	18.45	691.56	41.54			22.07
260	2367	8.30	6351	29.40	266.50	100.48			35.78
300	2697	7.72	5044	37.11	1568.71	124.49			77.9

Table S3. Parameters of the exponential fitting of the PL decay traces, and the average PL lifetimes of $FAPbBr_3$ NCs obtained from the traces in Figure 2c:

Temperature (K)	A_1	τ_1 (ns)	A_2	τ_2 (ns)	τ_{AVG} (ns)
4	11386	0.53	224	5.98	1.52
20	8251	0.68	709	7.59	4.07
40	7433	1.01	292	7.99	2.66
60	9956	0.96	380	7.43	2.44
80	9253	1.10	431	6.95	2.43
100	9272	1.39	632	7.57	3.06
120	9184	1.69	896	7.79	3.58
140	9060	1.98	1526	7.65	4.21
170	8701	2.57	1962	8.78	5.27
200	8394	3.21	2502	11.50	7.49
230	6450	3.51	4712	11.05	8.76
260	5009	3.78	5897	13.92	12.02
300	4136	4.12	6342	17.08	15.31

The temperature dependence of the PL linewidth can be described by the following equation[4]:

$$\Gamma(T) = \Gamma_0 + \Gamma_{AC}T + \Gamma_{LO}\left[exp\left({}^{-E_{LO}}/_{K_BT}\right) - 1\right]^{-1}$$

Where Γ_0 is the inhomogeneous broadening, Γ_{AC} and Γ_{LO} describe the coupling strength to the acoustical and LO phonons, respectively, and E_{LO} is the energy of the LO phonon. K_B is the Boltzmann constant. The values obtained from the fitting shown in Figure S6 are summarized in Table S4.

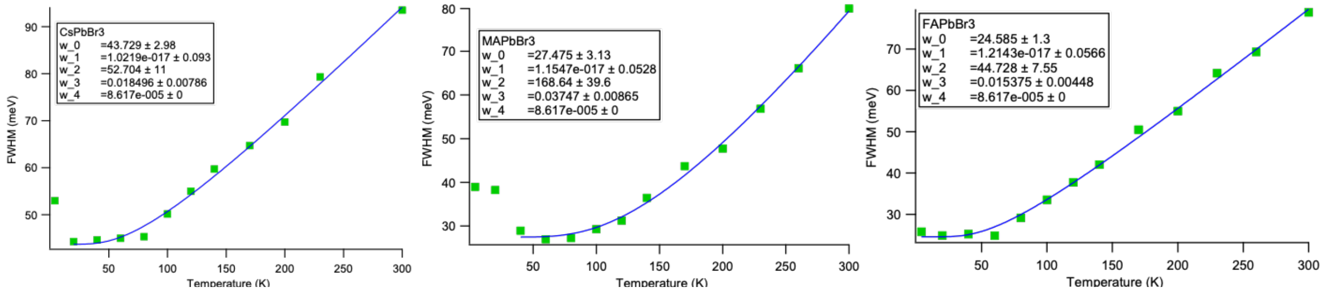

Figure S6. Fitting of the temperature dependence of the PL linewidth of $CsPbBr_3$ (a), $MAPbBr_3$ (b), and $FAPbBr_3$ (c) NCs with equation S1.

Table S4. Parameters obtained from the fitting in Figure S6.

**NC composition**	Γ_0 **(meV)**	Γ_{AC} **(meV)**	Γ_{LO} **(meV)**	E_{LO} **(meV)**
$CsPbBr_3$	43.7± 3.0	0 ± 0.09	53± 11	18.5 ± 7.9
$MAPbBr_3$	27.5 ± 3.1	0 ± 0.05	169± 40	37.5 ± 8.6
$FAPbBr_3$	24.6 ± 1.3	0 ± 0.06	45 ± 8	15.4 ± 4.5

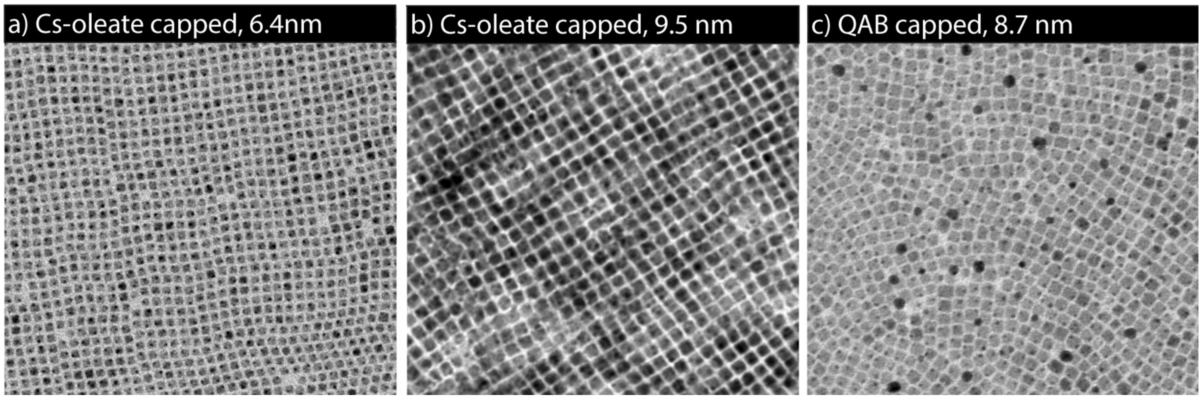

Figure S7. (a,b) TEM images of Cs-oleate capped CsPbBr3 NCs with different size, and of QAB passivated NCs obtained by ligand exchange.

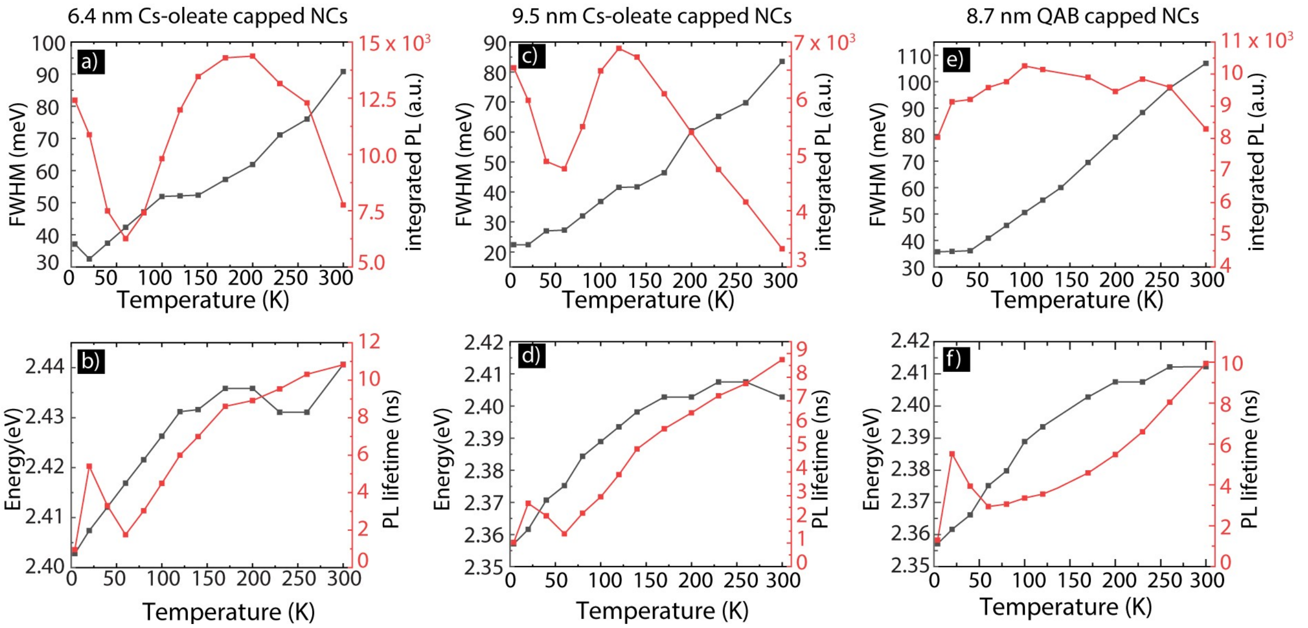

Figure S8. PL linewidth, intensity, peak position and average life time extracted from the spectra in Figure 4 (d-f).

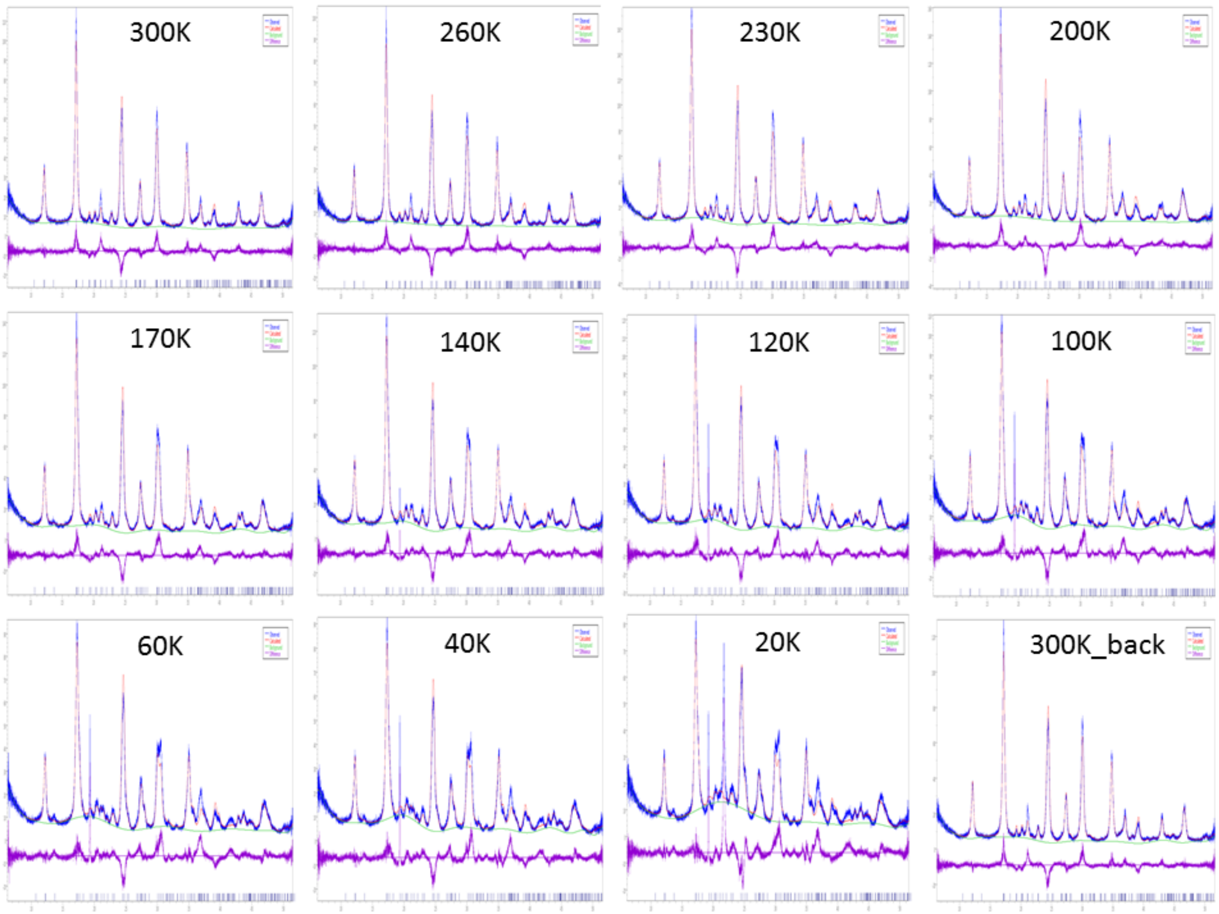

Figure S9. XRD spectra recorded at different temperatures for mixed ligands capped $CsPbBr_3$ NCs. We observed the appearance of additional sharp peaks from 140K down to 20K, which disappeared when the NCs film was heated back to 300K. We attribute these peaks to the formation of a thin ice layer at the sample surface after few hours of measurements at a pressure of at 10^{-6} mbar, or to frozen organics.

Table S5: Thermally induced variation in the cell parameters of mixed ligands capped $CsPbBr_3$ NCs

T (K)	a	b	c	Cell Volume
300	8.25906	8.28831	11.80331	807.98
260	8.22196	8.29093	11.78364	803.26
230	8.1856	8.27942	11.75434	796.62
200	8.1567	8.28185	11.73239	792.55
170	8.13283	8.28185	11.71081	787.92
140	8.10704	8.27305	11.68789	783.91
120	8.07976	8.2567	11.65699	777.66
100	8.08407	8.26566	11.66956	779.76
60	8.08502	8.29994	11.68757	784.3
40	8.05959	8.27552	11.65278	777.21
20	8.067	8.30508	11.70342	784.1
17	8.06812	8.3067	11.69584	783.85
300	8.25849	8.28184	11.80030	807.09

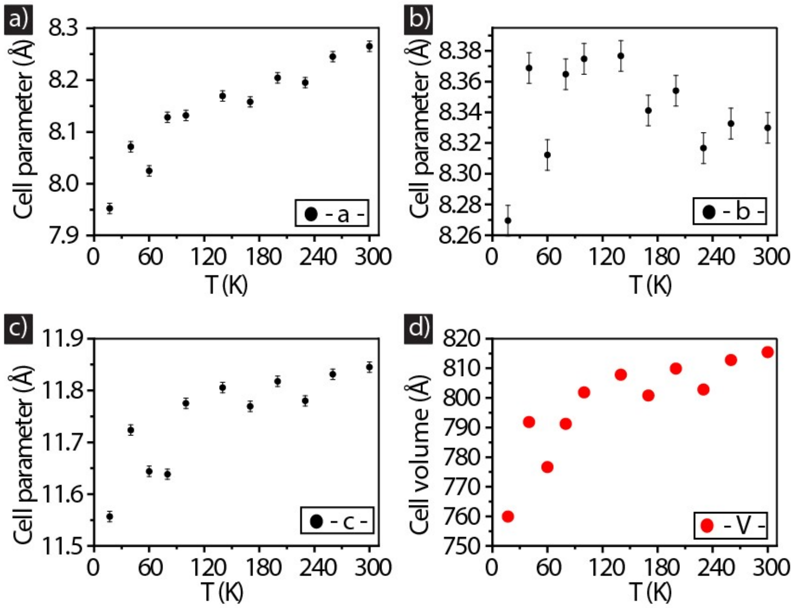

Figure S10: Cell parameters (a,b,c,V) obtained from the temperature dependent XRD measurements of Cs-oleate capped $CsPbBr_3$ nanocrystals. The data yields a thermally induced expansion in unit cell while retaining the orthorhombic phase.

References:

(1) Imran M, Caligiuri V, Wang M, Goldoni L, Prato M, Krahne R, De Trizio L, Manna L. Benzoyl halides as alternative precursors for the colloidal synthesis of lead-based halide perovskite nanocrystals. *J. Am. Chem. Soc.* **2018**, 140, 2656-2664

(2) Imran M, Ijaz P, Baranov D, Goldoni L, Petralanda U, Akkerman Q, Abdelhady AL, Prato M, Bianchini P, Infante I, Manna L. Shape-Pure, Nearly monodispersed $CsPbBr_3$ nanocubes prepared using secondary aliphatic amines. *Nano Lett.* **2018**, 18, 7822-7831

(3) Imran M, Ijaz P, Goldoni L, Maggioni D, Petralanda U, Prato M, Almeida G, Infante I, Manna L. Simultaneous cationic and anionic ligand exchange for colloidally stable $CsPbBr_3$ nanocrystals. *ACS Energy Lett.* **2019**, 4, 819-824

(4) Woo, H. C.; Choi, J. W.; Shin, J.; Chin, S.-H.; Ann, M. H.; Lee, C.-L. Temperature-dependent photoluminescence of $CH_3NH_3PbBr_3$ perovskite quantum dots and bulk counterparts. *J. Phys. Chem. Lett.* **2018,** 9, 4066-4074
